# Redox and Nitrosative Signaling and Stress

**DOI:** 10.3390/antiox9121237

**Published:** 2020-12-07

**Authors:** Claudia Penna

**Affiliations:** Department of Clinical and Biological Sciences, University of Turin, 10043 Torino, Italy; claudia.penna@unito.it

In this Special Issue, redox/nitrosative signaling has been considered in several aspects of cardiosciences and oncology, namely cardioncology. In particular, the authors focused on the role of physiologic levels of radicals, reactive oxygen species (ROS), and reactive nitrogen species (RNS). Besides considering the modulation of several physiological mechanisms and functions, the authors stressed that ROS/RNS can be involved in a plethora of pathological conditions. Indeed, ROS/RNS may lead to several diseases, such as cancer, neurodegenerative disease, lung disease, cardiovascular diseases, and ischemia/reperfusion injury. In an elegant review of this issue by Mailloux [1], the fundamental role of mitochondria in ROS production is considered. An important point stressed by the author is the importance of sex differences as a pivotal factor in determining cell redox buffering capacity. Indeed, the author reports evidence that mitochondria from the livers of female animals display higher rates of state 3 respiration, cardiolipin content, and increased oxidative phosphorylation following consumption of a high-fat diet [2]. Other important aspects of redox system were considered by Micera et al. [3]. These authors report the importance of squalene redox-related aspects in the sterol pathway. Indeed, squalene is an important metabolite involved in the regulation of many biochemical pathways, including the regulation of sterol physiology. In this review, the authors underline the role of squalene in the cardiovascular diseases (CVDs) associated with oxidative stress and suggest that several risk factors of CVDs can be treated with squalene. Notably, it was observed that a squalene-enriched diet was able to induce an enhancement of antioxidant systems against ROS/RNS burst. This finding was also stressed in the recent systematic review by Ibrahim et al. [4]. Other intriguing effects of ROS/RNS on cardiovascular pathophysiology were discussed in the review by Mercurio et al. [5]. These authors considered the role of the redox system in modulating the levels of tolerance to cardio-toxic anticancer drugs, focusing on redox aspects of ageing and metabolic alterations. In particular, the authors focused on CVDs and discussed the connections among CVD risk factors, cancer, and cardiotoxicity from anticancer drugs. Among risk factors, smoking, ageing, and metabolic syndrome including hypertension, obesity, and hypercholesterolemia were analyzed in depth. Indeed, alterations in ROS/RNS may have pivotal roles connecting malignancies and CVDs, as both cancer and CVDs are characterized by chronic inflammation and augmented ROS/RNS production, which may be interconnected [6]. The enhanced production of ROS has been linked to the risk of developing, among others, arterial hypertension and obesity, a condition that can be induced by anticancer treatments, leading to cardiotoxicity [7]. In an interesting original paper, Santos-Rosendo et al. [8] present experimental data relative to different oxidative markers in full-term placental villous obtained from normal weight and obese women, and from women with and without metabolic complications throughout gestation. The authors demonstrate a significant reduction in the levels of thiobarbituric acid reactive substances (TBARS) and carbonyl groups in full-term placenta. The authors conclude that a placental inactivation of antioxidant system response would lead to elevated oxidative stress in obese women during pregnancy. In another original paper, Park et al. [9] report interesting data regarding the protective effects of dauricine, an isoquinoline alkaloid, in bone. The results demonstrate that dauricine abolished the inflammatory action of LPS with a subsequent reduction of redox stress. Oxidative aspects of circuits regulating superoxide and nitric oxide were reported by Bil et al. [10] in an original paper. The authors have studied in several cell types from different tissues, including cancer cell lines, the expression of genes coding for enzymes involved in the regulation of cellular redox status. They demonstrate that cells have developed systems to maintain stable ROS levels in order to counteract oxidative stress. The authors suggest that different cell types use different redox pathways to cope with redox stress. It seems that some cells have special mechanisms that are triggered by high doses of radiation. Identifying the mechanisms by which cancer cells adjust to and survive in an oxidative environment may be important in radiotherapy. All of the articles in this Special Issue demonstrate that establishing a better understanding of oxidative stress is of pivotal importance in cardio-oncology.
